# Mistrust in government and National Health Insurance: A qualitative study of solo private practitioners in Cape Town

**DOI:** 10.4102/safp.v65i1.5768

**Published:** 2023-12-15

**Authors:** Bridget L. Perrow, Helen Schneider

**Affiliations:** 1 School of Public Health, Faculty of Community and Health Sciences, University of the Western Cape, Cape Town, South Africa; 2 SAMRC Health Services to Systems Research Unit, University of the Western Cape, South African Medical Research Council, Cape Town, South Africa

**Keywords:** independent private general practitioner, National Health Insurance (NHI), Universal Health Coverage (UHC), Cape Town Metropolitan Municipality, primary health care (PHC), public and private sector collaboration, stakeholder engagement, equitable healthcare

## Abstract

**Background:**

The participation of independent private general practitioners (GPs) is of fundamental importance to the successful implementation of key elements of the proposed National Health Insurance (NHI) reform, notably the contracting units for primary health care (CUPS). This study explored knowledge and perceptions of the NHI reforms of private GPs following the tabling of the NHI Bill in parliament in 2019.

**Methods:**

An explorative qualitative research methodology was adopted. Using a semi-structured guide, nine solo private GPs, purposefully selected to represent the range of practices in the southern peninsula of Cape Town were interviewed in depth by B.L.P. over the period from January 2021 to March 2022.

**Results:**

The GPs indicated support for the values of greater equity outlined in the NHI proposals. However, they had little engagement on or knowledge of their potential future roles in NHI. Concerns over financial viability and design were underpinned by an overall mistrust in the public sector to implement and manage NHI.

**Conclusion:**

The study concurs with previous research that private GPs are broadly in support of the principles of, and are potential allies, in advancing NHI. General practitioners need a platform to share their concerns and contribute as co-designers of NHI reforms. In the interim, steps to increase collaboration between private and public sectors at local and provincial level through, for example, referral processes may help to build the trust that is necessary between the sectors.

**Contribution:**

This study foregrounds the role of trust relationships in advancing NHI.

## Introduction

Promoting and safeguarding health is integral to a country’s well-being and sustainable economic and social development. This principle was agreed upon in the Alma-Ata Declaration of primary health care (PHC) in 1978, more than 40 years ago.^1^ These commitments and the 2030 Agenda for the Sustainable Development Goals (SDGs) were reaffirmed at the Global Conference on PHC in Astana, Kazakhstan in October 2018. The Declaration of Astana, adopted at the conference pledges to make bold political choices for health across all sectors, build sustainable PHC, empower individuals and communities and align stakeholder support to national policies, strategies and plans.^2^

A well-functioning health financing system is needed to achieve this. In 2005, member states of the World Health Organization (WHO) committed to develop their health financing systems to allow all people access to health services, without suffering financial hardship.^3^ This is known as Universal Health Coverage (UHC). Primary health care is at the heart of UHC.^2^

South Africa currently spends 8.9% of its gross domestic product (GDP) on health, which is distributed inequitably between public and private health sectors. Public health expenditure is just under 50% of total health expenditure, for 82.6% of the population.^4^ The cost of private specialist and hospital care has risen more than the consumer price index and skilled human resources are distributed heavily towards the private sector, while the public sector has remained poorly resourced.^5^

To address the inequities of this two-tiered system and move the country towards the realisation of UHC, the South African government has proposed the National Health Insurance (NHI) as a new financing mechanism. The NHI is premised on the need for transformation of current healthcare delivery where those with the most need have the least access to health services.^6^

National Health Insurance is intended to roll out over a 14-year period. The White Paper^7^ outlined the three phases of NHI implementation. Phase 1 (2012–2017) included piloting of health system strengthening focused on PHC, phase 2 (2018–2022) is concerned with legislation and the necessary structures of the fund and phase 3 (2023–2026) will complete the implementation of health system strengthening initiatives and introduce mandatory payment to NHI through taxes. The National Health Insurance Bill^8^ lays out the establishment of NHI.

An essential element in strengthening PHC proposed by NHI is enhancing capacity of the health system through contracting-in and contracting-out of private health practitioners. Contracting-in aims to reduce patient-overload in public health facilities. Contracting-out of PHC services requires that multi-disciplinary practices be configured into horizontal networks that are contracted through the contracting unit for primary health care (CUPs).^7^ The NHI will finance healthcare by entering into contracts with private or public facilities that have been accredited by an independent body, the Office of Health Standards Compliance (OHSC). The successful implementation of the NHI, aimed at building a unified and equitable system, requires the buy-in and co-operation of private general practitioners (GPs) who are intended to be key stakeholders in CUPs.

National Health Insurance in South Africa is a strategy to move the country towards UHC and is underpinned by the SDGs related to health.^9^ Solidarity and universality are key principles of the reform. However, McIntyre^10^ suggests that as an essentially redistributive policy, debate with the different stakeholders who have vested interest in the current system is to be expected, and emphasises that:

[…*P*]rogress in addressing the serious efficiency and equity challenges facing the South African health system can only happen if the public conversation focuses on how best to achieve a Universal Health System. (p. 17)

South Africa’s response to the coronavirus disease 2019 (COVID-19) pandemic, including the introduction of an extensive vaccination programme, suggests that the private and public sectors can work together. However, there is a need to build trust between the private and public sectors, recognising that this takes time and will require a combination of macro, meso and micro level strategies.^11^

Research on GP perceptions towards NHI in South Africa suggests a perceived lack of dialogue and consultation by the Department of Health on the implementation of the policy. In his study in the Western Cape, Valley^12^ highlighted poor communication between the provincial health department and the private GPs as a primary concern. In a recent study of GP perceptions towards NHI in the Chris Hani District, Gaqavu and Mash^13^ reported GP uncertainty around the government’s capacity to implement the policy and implications for solo GPs. The GPs also voiced a need for more information and engagement by government. Mathew and Mash^14^ conducted a similar study in the Western Cape and recommended that dialogue and collaborative engagement were needed to alleviate GPs’ fears and uncertainty around the NHI. They also recommended further research to understand how private GPs and practice groups wish to engage with policymakers in the future.

This study sought to explore the current perceptions towards NHI of a group of independent private GPs practising in the Cape Town Metropolitan Municipality, with the aim of eliciting their suggestions for policymaker engagement on NHI. As the study also took place during the COVID-19 pandemic, it provided a unique opportunity for the researcher to explore perceptions of this group of GPs towards NHI in an altered environment which required urgent collaborations between public and private sector to address the healthcare crisis.

The aim of the study was to explore how private independent solo GPs in the Western Cape viewed the introduction of the NHI and their role in it, following the tabling of the NHI Bill in the South African Parliament in 2019.

The objectives of the study were to explore the GPs’:

- knowledge of the NHI reform process and the provisions of the NHI Bill
- perceptions of the potential benefits, challenges and threats of the NHI Bill to their practices and their current systems and capacity to adapt
- experience of their engagement with the public sector and role players in the reform process to date
- vision on how communication may be improved between themselves and the Department of Health with regard to the implementation of the NHI.^11^

## Research methods and design

### Study design

The study adopted an explorative qualitative methodology, guided by grounded theory and interpretive in nature, based on the assumption that reality is socially constructed and that the researcher will bring their own values to the study.^15^

### Setting

The study focused on solo GPs operating in the Cape Town Metropolitan Municipality in the Western Cape province. Approximately 76% of the population of the Cape Metropolitan is uninsured,^16^ receiving free public primary healthcare through provincial and city clinic services organised into eight sub-districts. The remaining insured (24%) population and the uninsured willing to pay out of pocket make use of private primary care providers. According to a private sector database, there are approximately 1848 private GPs practising in either group or solo practices in the City of Cape Town.^17^ Depending on where they practise, these private GPs provide primary health care to a varying ratio of insured to cash patients. Some practices are licensed to dispense drugs, others not. Referral to either private or public sector facilities for higher levels of care is determined by the financial means and insurance status of their patients.

### Study population and sampling strategy

The study population comprised of private independent GPs in the Cape Town metropolitan and in particular GPs known to the researcher to be practising as solo practitioners.

General practitioners were sampled purposively,^18^ adopting the following criteria: firstly, respondents who were likely to be ‘information rich’, namely willing to share their honest opinions on their perceptions on NHI. Secondly, to represent the diversity of populations served by GP practices in the Cape Town metropolitan. From the GPs known to her, B.L.P. purposively selected 12 independent GPs of different ages and levels of experience, genders and populations served. Three GPs who initially agreed to participate in the research dropped out due to time constraints and nine GPs were finally interviewed.

Participants were recruited in a two-stage process. The researcher first contacted the GPs in person explaining the purpose and nature of the study, answering any questions they had, and explaining that the interview would be audio-recorded. Once the GP agreed to participate, signed consent was obtained. The researcher then arranged a suitable date and time to conduct the interview with the GP.

### Data collection

Participants were interviewed in person, via video chat or telephonically while they were at their private GP practices and at a time convenient to them. The interviewer was alone with the participants during the face-to-face interviews. The researcher used a semi-structured interview guide exploring ‘what’, ‘why’ and ‘how’ questions related to GPs’ knowledge and perceptions on the NHI and how communication and collaboration between themselves and policymakers can be improved. The guide was refined between co-authors after the first three interviews were completed. Field notes were kept documenting important practice information immediately after the interview. Notes were recorded about each GP practice based on the researcher’s prior knowledge. B.L.P. debriefed with H.S. following the interviews.

### Data analysis

Each audio-recorded interview was transcribed verbatim by the researcher and checked for errors. Once transcribed, the researcher analysed the data based on Burnard’s^19^ model of thematic content analyses. Notes were made during and after each interview of the interviewer’s impressions relating to the main topic. In a first stage of data immersion, transcripts were read carefully, and notes were made on emergent themes. Open coding was then undertaken to identify categories that covered most of the data. The number of categories was reduced and a final list of categories without overlap was produced. This list was validated by the second author (the study supervisor). The transcripts were checked again to ensure that all categories had been identified. A Microsoft Excel spreadsheet was drawn up to organise the coded sections into categories with sub-headings. This was completed by B.L.P. with regular consultation with the study supervisor (H.S.). At this stage, the writing up of results began.^11^

### Ethical considerations

The researcher informed the participants of her intent and rationale for conducting the study verbally and via an information sheet. Informed written consent was obtained before the interview was recorded. Participants were advised that they could withdraw from the study at any stage of the interview without consequences. The researcher ensured that there was no pressure on the GPs to participate in the study. There was no monetary or other incentive offered to participants. ‘Ethical approval to conduct the study was obtained from the University of the Western Cape’s Biomedical Research Ethics Committee (Ethics Reference Number: BM20/8/10) as partial fulfilment of a Master of Public Health Degree.

## Results

### The study participants

A sample of male (*n* = 5) and female (*n* = 4) respondents participated (Table 1). The GPs’ ages ranged from 30 years to 72 years. All the GPs interviewed were solo practitioners and represented a mix of predominantly cash paying and predominantly insured practices. Two of the GPs (P4 and P5) did sessions in local public sector hospitals and one (P9) did regular sessions assisting in theatres in a nearby private hospital. P8 did not own her practice and was practising in a sessional capacity for an independent private GP. P8 was the only participant who shared practice space, with a registered nurse in this instance.

**TABLE 1:** Participant profiles.

GP	Gender	Age	Dispensing	Patient profile (majority)	Public sector sessions
P1	Male	50–59	No	Cash	No
P2	Male	60–75	Yes	Insured	No
P3	Male	60–75	Yes	Insured	No
P4	Female	50–59	Yes	Insured	Yes
P5	Male	50–59	No	Insured	Yes
P6	Female	40–49	No	Insured	No
P7	Male	50–59	No	Cash	No
P8	Female	25–39	No	Cash	No
P9	Female	60–75	Yes	Insured	No

## Emerging themes

In analysing the interview transcripts, several common themes emerged, summarised in Table 2 in four main categories, namely (1) knowledge of the NHI Bill, (2) potential benefits of the NHI policy, (3) concerns over the NHI policy and (4) recommendations for stakeholder engagement and public-private collaboration.^11^

**TABLE 2:** Areas of enquiry and emerging themes from the data.

Area of enquiry	Emerging themes
Knowledge of the NHI Bill	Knowledge gap on role of the independent private GP in relation to the existing public primary care facilities and the proposed referral system
Potential benefits of the NHI Policy	The provision of equitable healthcare Potential to advance the primary health care service Increased accountability for private independent GPs
Concerns with the NHI Policy	Financial viability of NHI in South Africa Fear of corruption and nepotism Concerns regarding management and administrative capabilities Design concerns
Recommendations for stakeholder engagement and public-private collaboration	Provide opportunities to share experience and skills Further input on pilot projects and clarity on NHI policy areas affecting independent private GPs needed Information sharing and referral between private and public sectors

NHI, National Health Insurance; GP, general practitioner.

### Knowledge of the National Health Insurance Bill

The researcher enquired about how the private GPs communicate with the public sector in general. These enquiries were followed up with a question to understand how the GPs believed the channels for this communication could be facilitated. The GPs reported attaining their information on NHI from a variety of sources. These included independent practitioner associations (IPAs), medical associations, social media or other Internet research. None had received any direct formal communication from the Department of Health regarding NHI proposals. Nevertheless, through their own research, feedback from their IPA, continuing medical education (CME) meetings and medical funders, the GPs had a fair overall understanding of the proposed NHI, as it stands:

‘I think it sounds fantastic in theory. My concerns will be in the implementation. How is it going to work, what role will GPs play and how will we be funded by the government? Those are my concerns. I do feel that it is good that the government is looking at more holistic ways to treat everyone in the country but integrating the private and state sectors is going to be challenging.’ (P8, female, 25–39 years old)

However, most were uncertain about their actual role in the reformed health system once NHI was implemented, including the distribution of roles between the GP and the existing public sector clinics, and the proposed referral system:

‘The one thing I don’t understand about the NHI is what is going to happen to the primary healthcare facilities? Yes, how are they going to work? If they keep them and patients are allowed to go to ordinary GPs aren’t those places going to be empty?’ (P1, male, 50–59 years old)

Several GPs expressed uncertainty over how the apparent shift from solo independent GP practices to group practices will be managed.

### Potential benefits of the National Health Insurance

Despite several concerns, all the GPs interviewed in this study expressed their approval of reform that aims to provide equitable healthcare. There was an acknowledgement that the existing healthcare system is not sustainable, and that reform is necessary.

‘I think we definitely should have an NHI because we need to have access to medical care, um, for people of all walks of life, from rich to poor. And as things stand when the NHI does come in with its teething problems and it is never perfect, it should hopefully be better than the system that we have now. People are unable to access healthcare. Whether it works as planned, that is another thing, another story. But the idea of NHI is a good one.’ (P4)

Many of the GPs highlighted the benefit of prioritising PHC through the reform process.

‘Primary care is the basis for this and I would be very keen to be part of that primary care set up.’ (P2)

Finally, the need for increased accountability of private GPs was mentioned. Several GPs were of the view that the proposed reforms had the potential to ensure that this is achieved:

‘When people are in a solo practice as I mentioned to you before, people can pretty much carry on in their own way and it may become a case of repeating bad habits that become your norm so I think in terms of keeping up to date also group practices or district hospitals will be the ideal way to ensure the quality of care we are practicing is acceptable.’ (P4, female, 50–59 years old)

### Concerns with the National Health Insurance

The affordability of NHI within the South African context was a key concern for the GPs:

‘So, my first concern is how are these huge amounts of money are going be accessed having a vast discrepancy between the have and have nots. Those, a small number of people who are employed and a vast number who are unemployed.’ (P2, male, 60–75 years old)

The vested interests of stakeholders such as the dominant medical aids and private hospital groups were also raised as a potential threat to the successful introduction of NHI. As a result of their doubts of financial viability and vested interests, several GPs did not believe that the NHI would be introduced while they were in practice:

‘Basically, zero as I do not see the NHI happening in my time.’ (P4, female, 50–59 years old)

Others had sharper concerns over the potential for corruption and nepotism within NHI:

‘I feel very worried, very worried about the roll out, about how the NHI is going to work and the reason I feel that way is that the public sector is rife with corruption and is poorly managed from the finance system perspective.’ (P8, female, 25–39 years old)

The GPs in this study expressed support for the proposed capitation remuneration, and there was consensus that this was more favourable than a fee-for-service payment system. However, they also regarded this payment system as being open to risks, possibly leading to excessive patient volumes and impacting on the quality of care that they could provide:

‘I don’t know the details of what the amount is that they will be proposing. But for example, with managed health care that is currently available, I have removed myself from those because we have found that they are too restricted in what care you can actually provide the patients with in terms of access to hospital care, where you can send them, what medicine you can prescribe. I think doctors are overwhelmed as they reduce the payment to the doctor but give a stipend each month for each patient on your books even when they don’t come. But when they do come, they come at a reduced rate and come as often as they want so it [*is*] impossible to manage the patients and you actually can’t provide good medical care.’ (P4, female, 50–59 years old)

Further design concerns related to access to healthcare services by foreign nationals under the proposed NHI system, and choice of where GPs could practise in future. Although loss of autonomy was raised, it was not a primary concern of most of the GPs.

### The impact of the COVID-19 pandemic on this general practitioner community

The researcher kept a diary and recorded notes of observations made following initial practice visits to arrange the research interviews. It became very clear that the circumstances resulting from the COVID-19 pandemic had had a profound impact on these private GPs, professionally and personally. They had dealt with the loss of their patients, colleagues and in some instance their friends and families. Several had spent significant time in hospital themselves due to COVID-19. Struggles reported by GPs included difficulties screening patients attending their practices, working in protective gear, managing anxiety in patients and their relatives, many administrative and financial challenges and difficulties balancing personal and professional demands. It was notable that despite a sense of practising in isolation during this time, there were GPs who reported being encouraged by collaborations between the public and private sectors during the crisis. Particular reference was made to the prioritisation of intensive care treatment facilities and the national COVID-19 vaccination programme rollout.

### Recommendations for stakeholder engagement and public-private collaboration

While several of the GPs did not believe that NHI would materialise soon, they expressed a willingness to share their skills and experience to contribute towards the implementation of NHI:

‘It is very important that they communicate with us. We have had no direct communication and often read about NHI on news websites but nothing directly. It is important because we have some input- because we are doing the work! After practicing for 40 years, we have seen the changes and we can see the need.’ (P9, female, 60–75 years old)

All the GPs wanted more information on recent NHI policy developments and felt there was an urgent need for improving collaboration between the public and private sectors. Practical suggestions included sharing patient test results and allowing tools for improved communication on patient outcomes regardless of whether they were seen in the private or public sector:

‘I wanted to say earlier what I think would be a really nice thing to do from now to allow communication between private and public sector in terms of allowing results to be shared. I am not sure how it would work in terms of POPI [*the Protection of Personal Information Act*] but for a clinic because they need a workup and cannot afford it in private the patient gets scolded for going to a private doctor even though they don’t have the capacity to look after all the people. And we don’t get the patient’s results. And the patient may say they never heard back and think everything is alright and you don’t know if things have fallen between the cracks, what the results were and what tests were done It ties your hands in how to manage the patient.’ (P4, female, 50–59 years old)

Several GPs interviewed in this study referred to the VULA (Virtual Unbundled Local Access) e-referral application as a useful tool to facilitate patient referrals to tertiary public health departments and the recommendation was made to consolidate the different systems:

‘For instance, if I want to refer a non-medical aid patient who needs expensive care in the area, you go on to a website and refer, and then you must scroll on and look for the site as there are several websites. Some of the departments are not on VULA. For example, the breast clinic. You have to search which makes it difficult. An easier platform where they are all in.’ (P9, female, 50–59 years old)

## Discussion

The solo private GPs interviewed in this study saw the need for healthcare reforms such as NHI and indicated their support for the values of equity, as outlined in the NHI proposals. They were in favour of design elements such as capitation payment systems. However, their attitudes were underpinned by a general organisational mistrust in the public health system and its capacity to ensure an effective and fair policy process. Mistrust stemmed from an ongoing failure of engagement, a recurring theme from prior studies, a lack of policy detail on how NHI would work in practice, and concerns over the financial viability and risk of corruption.

For the last 10 years, studies of GPs in the private sector have consistently raised the need for improved communication with the Health Department.^12,14,20,21,22,23^ In their study of GPs in the Oliver Reginald Tambo NHI pilot district (Eastern Cape), Hongoro et al.^24^ reported that although most doctors were interested in participating, ineffective communication contributed to the low uptake of the proposed national contract. Surender et al.^23^ similarly explored the views of 55 purposefully selected GPs participating in a pilot NHI contracting site in Tshwane. Despite strong support for the NHI principle, the doctors interviewed were sceptical that private doctors would embrace the proposed contracting scheme. The authors reiterated that if the government agenda for healthcare reform is going to be realised, there is a need for the Department of Health to be more engaged with GPs. The findings in this study are consistent with these earlier studies.^12^

Previous studies have raised concerns regarding accreditation and uncertainty around what practice requirements would mean for the GPs.^12,14,23^ Researchers also found that the capitation reimbursement model could result in higher patient volumes, compromising the quality of care.^14,21,22^ But In this study, the main concern expressed was a fear of the potential for corruption and nepotism through the introduction of NHI, which was underemphasised in earlier literature.

In a recent survey conducted by the Concentric Alliance and Section 27,^25^ a lack of trust was advanced as the primary reason for the current conflict around NHI, concurring with this study’s findings. Gilson^26^ argues that trust underpins the co-operation required within health systems, where trust is commonly understood to be ‘the optimistic acceptance of a vulnerable situation in which the trustor believes the trustee will care for the trustor’s interests’. Although trust is usually described in one-to-one relationships, it also has an organisational or institutional dimension, also referred to as generalised trust. Institutional trust may be affirmed or undermined by inter-personal relationships within systems.^27^

### Strengths and limitations

B.L.P.’s tacit knowledge of the GPs and established relationships may be considered as a strength of the study, especially in completing the recruitment process. Commencing this study, the researcher initially planned to interview a minimum of 12 participants, approached 20 potential participants, and finally succeeded in completing nine interviews amid the constraints of the COVID-19 pandemic. In total, six interviews were conducted face-to-face and three interviews were conducted remotely, with interviews lasting between 15 min and 45 min. The researcher encountered challenges recruiting the diversity in age within the GPs as most participants were above 50 years. Possibly, the mature GPs, especially those nearing retirement did not believe the policy would impact them directly. The younger GPs appeared to be far more apprehensive regarding the impact that the policy would have on their ability to practise privately. The researchers would have preferred a larger sample reflecting more diversity in age. However, we agreed that data saturation had been reached on the theme of trust.

### Reflexivity

B.L.P. qualified as a registered nurse (RN) (B Nursing) in 1997 and had practised in the Western Cape area as an RN initially and subsequently as a medical representative in various roles. Having consulted with private GPs in the area in a professional capacity as a medical representative for the past 12 years, the researcher has tacit knowledge of approximately 250 private independent GP practices within the Cape Town metropolitan. In her role as a medical representative, the researcher has established relationships with GPs practising in varying socio-economic situations. Although familiar with the GPs professionally, the researcher had limited experience conducting qualitative interviews. The researcher was also aware that her familiarity may have introduced a bias both in sampling and in the participant’s responses such as their attitudes towards equity in healthcare (social desirability). H.S. holds a Doctor of Philosophy (PhD) and has over 25 years of experience working on the problems of South Africa’s health system. H.S. supported B.L.P. throughout the research process.

Working with and being naturally sympathetic to the GP community necessitated considerable self-reflection and seeking a critical distance on the part of the researcher. The advent of the COVID-19 pandemic also required the researcher to adapt to an altered environment to collect the data, remaining mindful of the stress the GPs faced daily. Conducting remote interviews, rather than face-to-face, resulted in significantly shorter interviews which inhibited the exploration of the GPs’ perceptions. Despite the challenges that arose due to the pandemic, the researcher witnessed the resilience and tenacity of the GPs through this period.

Finally, the solo practitioners selected for the study may have been less informed and oriented towards future NHI arrangements such as the CUPs, compared to group practices.

## Conclusion

Despite the difficult environment in which this study was conducted, the findings support earlier research in this field, yet bring new perspectives for NHI policy development and suggested avenues to facilitate collaboration between the public and private sectors. In addition, this study provides some insight on the impact that the COVID-19 pandemic and emergency policies have had on this group of independent private GPs.

### Recommendations

Based on the findings of this study, the researcher recommends that among the landscape of private sector actors, GPs could be considered allies rather than ‘interests’ that need to be ‘managed’. Steps could be taken to include private GPs into the reform process through more direct means of communication and the acknowledgement of the contribution that they can make towards assisting in achieving the NHI. Developing platforms that enable interaction between decision-makers, implementers and GPs, will provide the added benefit of affording independent private GPs an opportunity to voice recommendations based on their knowledge and experience within the health sector.

The researcher recommends further research to gain greater understanding of alternative ways to develop stakeholder engagement with independent private GPs, especially for those in their early years of private practice. This group has recently been active in the public sector and may have valuable insights into possible ways to develop collaborative efforts between the sectors. Active steps to increase collaboration between private and public sectors through, for example, clear referral processes and results sharing were proposed by GPs in this study. These forms of co-operation between the two sectors may be ‘small wins’ that increase the sense of shared purpose and contribute to building the trust needed to advance NHI reforms.^11^
